# ECG Monitoring Garment Using Conductive Carbon Paste for Reduced Motion Artifacts

**DOI:** 10.3390/polym9090439

**Published:** 2017-09-11

**Authors:** Jin-Woo Lee, Kwang-Seok Yun

**Affiliations:** Department of Electronic Engineering, Sogang University, 35 Baekbeom-ro, Mapo-gu, Seoul 04107, Korea; jinnnnu@sogang.ac.kr

**Keywords:** electrocardiogram, wearable, graphite paste, garment

## Abstract

The heart is a fundamental organ of the human circulatory system and the continuous measurement of electrocardiogram (ECG) signals is of great importance for pre-detection of heart diseases. Dry electrodes that do not require electrolyte gel have been developed for wearable ECG monitoring applications. However, this kind of electrode often introduces motion artifacts because of the high contact impedance between the electrode and skin. We propose a wearable ECG monitoring garment that employs electrodes made of conductive carbon-based paste. This paste is directly applied to the skin and after drying for 5 min, it forms a patch electrode that is detachable and flexible. The contact impedance between the patch electrode and the skin is very low because the paste covers the skin in a conformal manner. The experimental results show that the contact area of the carbon-based paste on the skin replica is almost 100%. At frequencies under 10 Hz, the contact impedance of the patch electrode is of 70.0 kΩ, much lower than the typical 118.7 kΩ impedance of a Ag/AgCl electrode. We also demonstrate that the ECG signals measured using the custom-designed garment and the patch electrodes are very stable even during actions such as walking and running.

## 1. Introduction

The rapid development of mobile technologies combined with a progressively aging population has resulted in the emergence of wearable health care devices that enable continuous monitoring of personal health anywhere and at any time [1,2,3,4,5]. This continuous monitoring makes it possible to prevent a disease or keep it safely under control and avoid emergency intervention. Currently, several wearable health care devices provide bio-signals, such as electrocardiograms (ECGs), electromyograms (EMGs), electroencephalograms (EEGs), body temperature, blood pressure, respiratory rates, and blood glucose levels [6,7,8,9,10].

Among the various bio-signals, ECG signals are one of the most important because they help monitor the electrical activity of the heart and return useful information about the heart condition and heart-related diseases, such as arrhythmia, cardiomegaly, premature atrial contraction, and premature ventricular contraction [11,12,13,14,15]. The electric impulse generated at the sinus node of the heart contracts the atrium and ventricles, enabling blood circulation in the body. The electric signals from the cardiac systole and diastole generate tiny electric currents that spread through the body and induce potential differences at different positions. Typically, an ECG signal can be recorded by placing three electrodes on the skin and sensing the potential difference between them. These signals are of very small amplitude, ~1 mV, and are affected by noise [16,17], therefore, requiring appropriate signal amplification and noise-reduction techniques. Additionally, ECG measurements made in dynamical conditions contain motion artifacts due to different causes: impedance changes, friction, high contact impedance, and change of half-cell potential induced by an unstable contact between electrodes and skin [18]. The motion artifacts are greatly influenced by the electrode-skin contact impedance which is, therefore, commonly used to assess the quality of ECG signals [17,18].

In the field of clinical and medical research, a standard commercial Ag/AgCl electrode is used to record bio-signals such as ECG, EMG, and EEG. This electrode employs an electrolyte gel for establishing a good electrical connection between electrode and skin and can provide a high-quality signal, but it has also several drawbacks. The electrolyte gel can irritate the human skin, and the signal quality degrades over time due to the dehydration of the gel [17,18]. For this reason, Ag/AgCl electrodes are not suitable for wearable health care devices which are intended for long-term continuous monitoring. To overcome these issues, many dry electrodes not requiring an electrolyte gel have been studied [19,20,21,22,23,24,25,26,27,28,29,30,31,32,33]. Among the proposed solutions, rigid metal electrodes are not suitable for wearable applications owing to their high contact impedance, and the presence of motion artifacts caused by variations in the contact area during body movements [19]. The contact impedance of metal electrodes can be reduced by using a micro-needle array structure [20], but this approach can cause severe damage to the skin due to penetration of the micro needles. Some of the recent approaches use flexible electrodes, such as textiles and conductive polymers, in order to increase the contact area between the skin and the electrode by making conformal contact, which reduces the contact impedance [21,22,23,24,25,26,27,28,29,30,31,32,33]. Textile electrodes can be used in a wide variety of applications and have a larger contact area compared to rigid electrodes, but do not provide completely conformal contact with skin [25,26,27]. In contrast, the conductive polymer, made from carbon and polymer, can make more effective contact compared to a rigid electrode and has a low contact impedance. However, motion artifacts are still present, and it is difficult to obtain an ECG signal in real-time during body motion [28,29,30,31,32,33].

**Figure 1:**
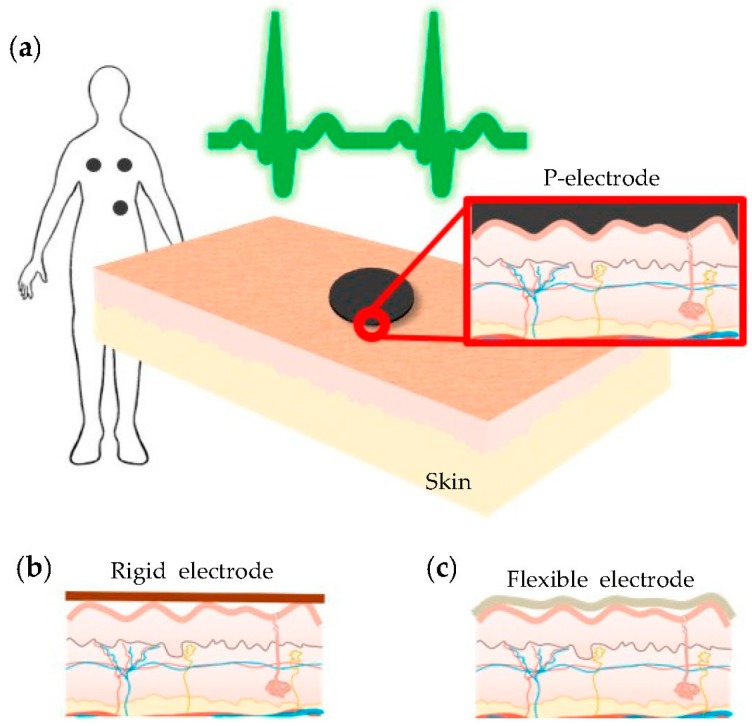
Schematic view of cross-section between the skin and various electrodes of (**a**) the proposed P-electrode; (**b**) a rigid electrode; and (**c**) a flexible electrode.

In this study, we propose a wearable ECG monitoring garment whose contacts are made of a conductive carbon-based paste (C-paste), which ensures low motion artifact generation and stable long-term monitoring. One of the key factors affecting the contact impedance is the contact area between the skin and the electrode [34,35]. Figure 1 shows a cross-section of the contact area between the skin and various electrodes: the proposed patch electrode (P-electrode), a rigid electrode, and a flexible electrode. Since the skin surface is rough, as shown in the figure, the contact area with a rigid electrode is very limited (Figure 1b). The flexible electrode also does not provide completely conformal contact with the skin, though its contact properties are better than those of rigid electrodes (Figure 1c). Instead, the electrode made of C-paste is directly applied to the skin and, thus, guarantees conformal contact with a low contact impedance. After application on the skin, the C-paste dries in a few minutes and becomes a P-electrode that offers good electrical conductivity and mechanical flexibility (Figure 1a). In addition, the P-electrode made of C-paste can be easily applied to any part of the skin and can also be easily removed by detaching it from the skin.

## 2. Materials and Methods

### 2.1. Preparation of C-Paste

The C-paste consists of graphite powder (150–200 mesh, Duksan, Ansan, Korea), polyvinyl alcohol (PVA), water, ethanol, and butylene glycol. PVA is used as a film former, making it easy to peel off the P-electrode from the skin. Ethanol is used to quickly dry the paste-like carbon electrode after its application on the skin. Graphite powder composed of fine carbon particles gives the P-electrode its good conductivity. The graphite powder is not toxic, but ultrafine carbon nanoparticles may cause unexpected harmful effects if they penetrate the skin. Therefore, we use graphite powder with particles that are 74–90 μm in size, larger than the average size of human skin pores, 47 μm [36].

The graphite powder (30 wt %), H_2_O (50 wt %) and butylene glycol (3.5 wt %) are mixed and dispersed by magnetic stirring for 1 h at 80 °C. Then, the PVA (8 wt %) and ethanol (8.5 wt %) mixture solution is added to the pre-dispersed solution and dispersed again by a magnetic stirrer for 1 h at 150 °C.

### 2.2. Preparation of Electrocardiogram (ECG) Measuring Garment

**Figure 2:**
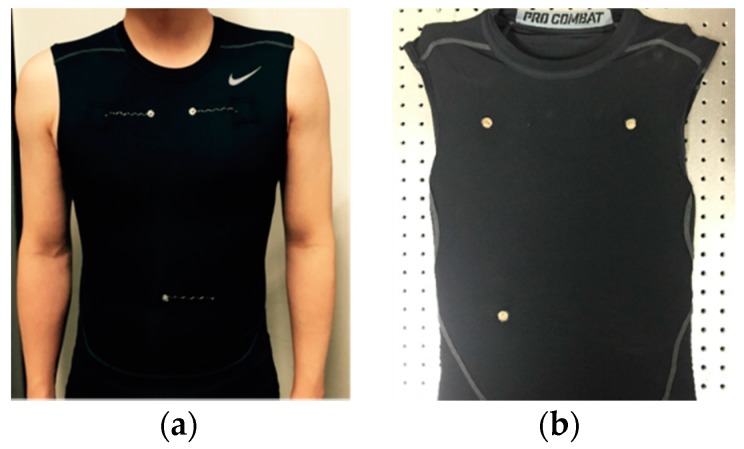
Electrocardiogram (ECG) measurement garment. (**a**) Outside of the garment; and (**b**) inside of the garment.

Figure 2 shows inside and outside of the ECG measurement garment. A tight-fitting, but elastic, shirt (Pro Combat T-shirt, Nike Korea, Seoul, Korea) is used as the test garment. Electrical contacts to the garment are provided by small disks of silver fabric (Medtex P-180, V Technical Textiles Inc., Palmyra, PA, USA) stitched inside the garment using a conductive thread (DEV-10118, Bekaert, Zwevegem, Belgium). The average sheet resistance and thickness of the silver fabric were measured to be 1.2 Ω/square and 0.5 mm, respectively. Then, a small amount of silver epoxy was applied at the interface of the silver fabric and the conductive thread to obtain reliable electric contact and low contact resistance, even during human motion. We used silver fabric with a diameter of 1 cm to compare it with a commercial Ag/AgCl electrode of the same size.

### 2.3. Measurement Setup

To measure the sheet resistance, the P-electrode was spin-coated on the silicon wafer with a thin Si_3_N_4_ layer on the surface and dried for 5 min at room temperature. Then, the sheet resistance was measured with a four-point probe and source meter (Keithley 2400, Tektronix, Beaverton, OR, USA).

We prepared a skin replica to quantify the contact area between human skin and several types of electrodes. The fabrication process of the skin replica begins by pouring Dragon Skin 30 (Smooth-on) on the skin and curing it 4 h at room temperature [33]. After peeling the cured Dragon Skin, a polydimethylsiloxane (PDMS) pre-polymer was poured on it and cured for 2 h at 65 °C. Finally, a transparent PDMS skin replica can be obtained by detaching it from the Dragon Skin mold. The contact area was measured by analyzing the pictures with the histogram calculator of Adobe Photoshop (Adobe Systems, CC 2015, San Jose, CA, USA).

**Figure 3:**
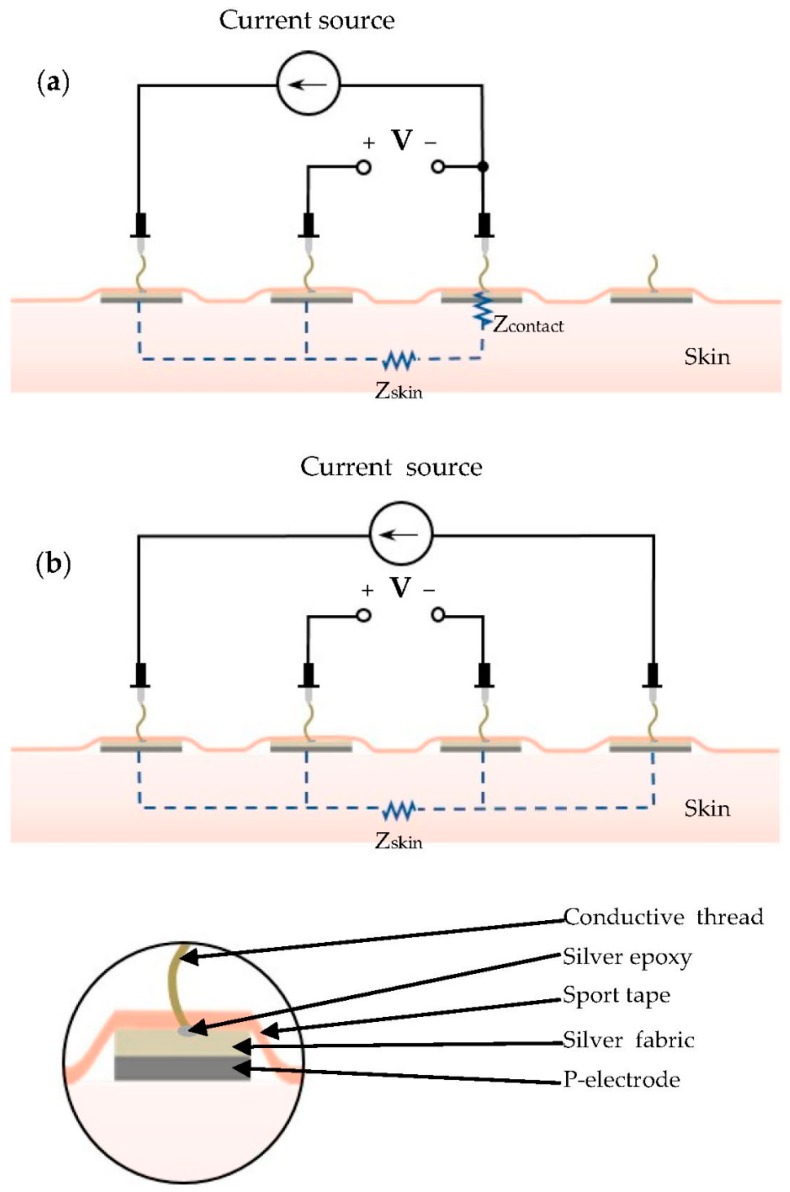
Experimental setup used to measure contact impedance. (**a**) Three-point probe configuration; and (**b**) four-point probe configuration.

The contact impedance between the human skin and P-electrodes was measured by using an impedance analyzer (Solartron 1260, Ametek, Berwyn, IL, USA). First, four dots of C-paste with a diameter of 20 mm were applied on four positions on the subject’s arm skin, and dried for 5 min to form P-electrodes. Then, silver fabric patches with a thickness and diameter of 0.55 mm and 10 mm, respectively, were placed on the P-electrodes and held in place firmly with sport tape. The measurement probes were connected to the four electrodes by means of conductive threads, as shown in Figure 3. The contact impedance was then analyzed by measuring the impedance between the electrodes [33]. First, the total impedance of Z_contact_ and Z_skin_ was measured by using the three-point probe configuration shown in Figure 3a. Then, the skin impedance, Z_skin_, was measured by using the four-point configuration shown in Figure 3b. Finally, the contact impedance, Z_contact_, was obtained by subtracting the skin impedance from the total impedance.

**Figure 4:**
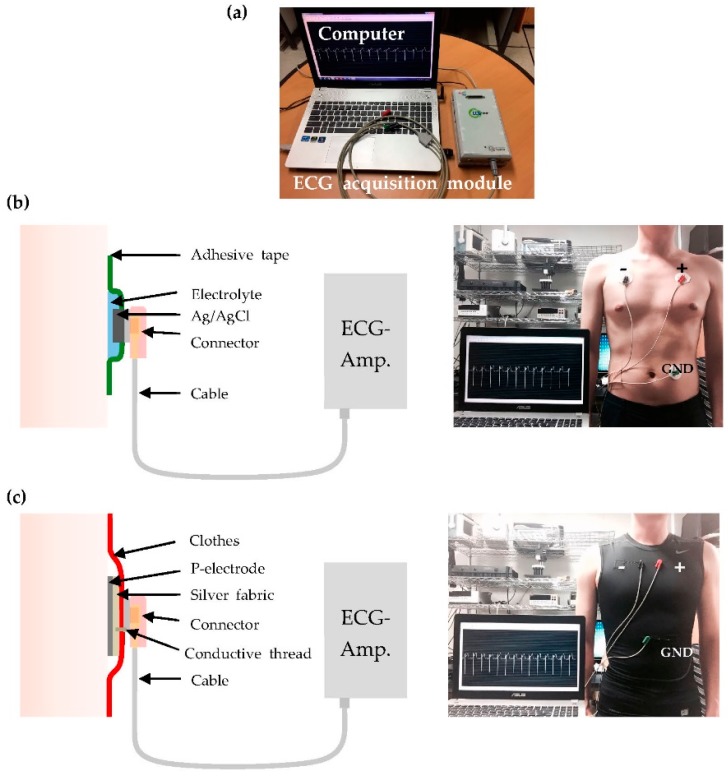
Experimental setup and configuration for ECG measurement. (**a**) ECG acquisition module, shielded cable, and computer; (**b**)the configuration of the experimental setup used for ECG measurement by using commercial Ag/AgCl electrodes; and (**c**) the experimental setup when using the proposed ECG measurement garment with P-electrodes on the skin.

Figure 4 shows the ECG measurement setup. The positive and negative electrodes are placed on the left and right of the chest of the subject, respectively. The ground electrode is positioned on the left part of the abdomen, as shown in the Figure 4b,c. The real-time ECG signal was measured using a data acquisition module (idaq-400, PhysioLab Co., Busan, Korea), an ECG amplifier (PhysioLab), and shielded cables (PhysioLab). One end of the shielded cable is a snap button-type connector to the Ag/AgCl electrode. The commercial pre-gelled Ag/AgCl electrodes were attached to the skin, fixed with adhesive tape, and subsequently connected to the ECG amplifier through the cables. A similar measurement configuration was used for the custom-designed ECG measurement garment. Silver fabric and metallic snap buttons were attached to the inner and outer surface of the cloth, respectively, and were stitched to one another using conductive thread. The snap button was connected to the ECG amplifier through the shielded cable, as shown in Figure 4c.

## 3. Results and Discussion

### 3.1. P-Electrode and Its Contact Properties with Skin

**Figure 5:**
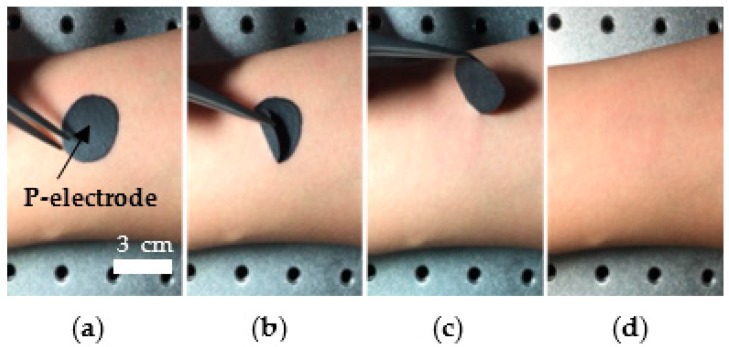
Easy removal of the P-electrode from the skin. (**a**) P-electrode formed on skin; (**b**,**c**) manual removal of the P-electrode from skin; and (**d**) the skin right after the detachment of the P-electrode.

The C-paste is fully dried and forms P-electrodes in five minutes at room temperature, if it is applied on solid surfaces, such as human skin, and exposed to air. The P-electrode can be easily detached from the skin as shown in Figure 5. After detaching the P-electrode, slight irritation and red color were observed on the subjects’ skin, which disappeared in a few minutes. No itching, swelling, or pain were observed, but further studies are required to identify the effects of the P-electrode on human skin. The sheet resistance of the P-electrode was measured in 10 different samples, and was found to be 165 Ω/square on average, which are 0.035 Ω·m in resistivity.

**Figure 6:**
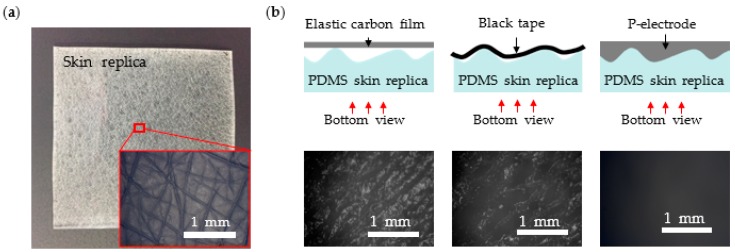
(**a**) PDMS skin replica and its microscopic structure; and (**b**) schematic cross-section views and microscopic pictures seen from the bottom surfaces for several contacts: flat P-electrode film, black tape, and P-electrode.

Figure 6a shows the fabricated PDMS skin replica and its magnified view. Figure 6b shows the schematic cross-section views for different electrodes and the actual microscope pictures of contact surfaces for electrodes made of elastic carbon film, black tape, and P-electrode. The grey zones in the pictures are due to air trapped between skin replica and electrode and, therefore, indicate the non-contacting area. A completely black picture, therefore, corresponds to the conformal contact between the skin and the electrode. We found that the contact area with the skin replica was 39.4% for the flat carbon film and 55.3% for the black tape. In contrast, the contact area of P-electrode is almost 100%. Since a gel-type C-paste is applied on the surface and dried in place, the electrode can achieve conformal contact even with roughly wrinkled skin.

**Figure 7:**
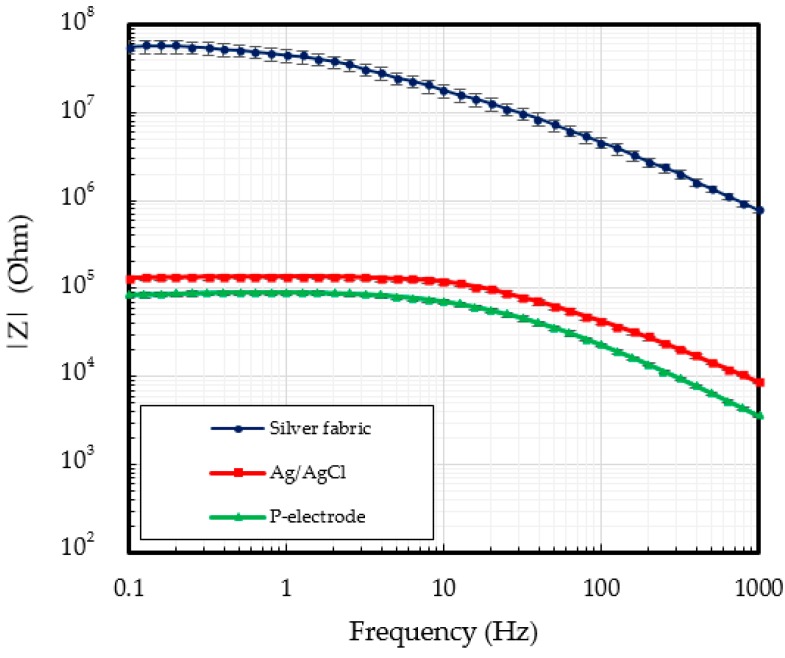
Contact impedance of the silver fabric, Ag/AgCl, and P-electrode.

Figure 7 shows the contact impedance as a function of the signal frequency. The contact impedance of silver fabric alone is too high to be used in ECG measurements. The measured contact impedance of the Ag/AgCl electrodes was of 118.7 kΩ for frequencies under 10 Hz. Due to the conformal contact with the skin, the lowest contact impedance of 70.0 kΩ was obtained for the proposed P-electrodes.

### 3.2. ECG Measurement Results

**Figure 8:**
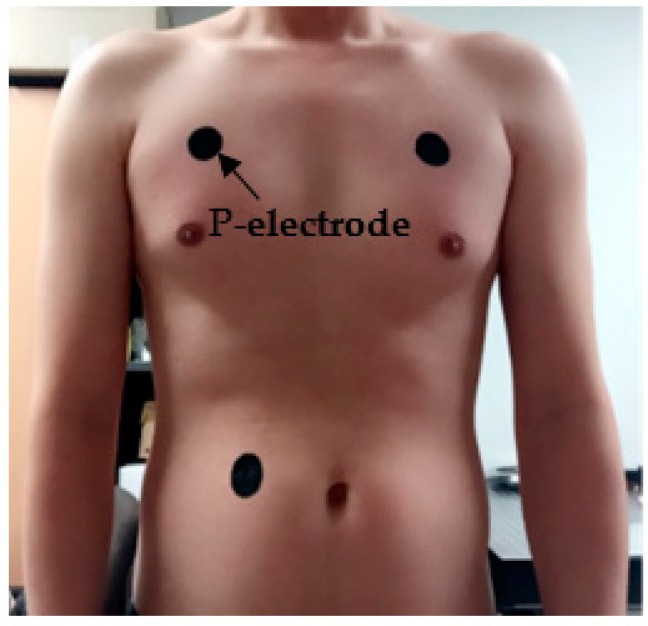
Application of P-electrodes on the subject’s body.

The ECG measurement using P-electrode was performed by wearing the ECG measurement garment shown in Figure 2, with the P-electrodes applied on three positions on subject’s body as shown in Figure 8. No additional actions have been taken to increase the contact between the garment and the P-electrodes.

**Figure 9:**
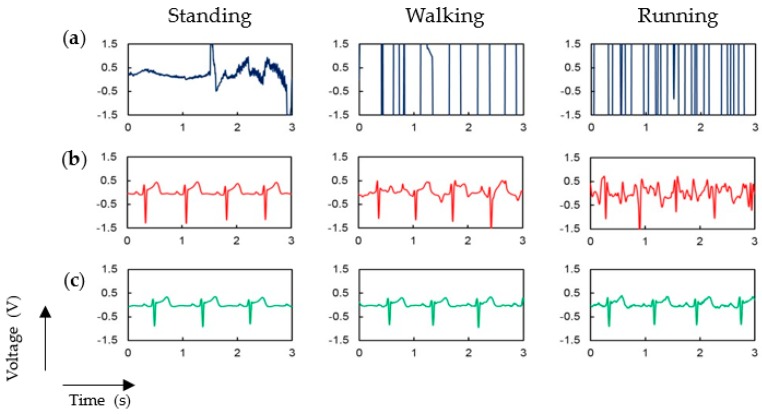
Measured ECG signal at three different levels of motion (standing, walking, and running) by using (**a**) ECG measurement garment without P-electrodes; (**b**) Ag/AgCl electrodes; and (**c**) ECG measurement garment with P-electrodes.

Figure 9 shows the ECG signals obtained during three activity levels: no motion in standing position, walking and running. As a comparison, we repeated the measurements in the same conditions with the same garment but without P-electrodes, and the ECG signal was not distinguishable at all even without body movement (see row (a) of Figure 9) because of the high contact impedance of silver fabric. Figure 9b shows the ECG signal measured by using commercial Ag/AgCl electrodes. In this case, a very stable signal was obtained if the subject is at rest. However, as the subject moves, the signal became unstable because the motion artifacts. The larger the body movement, the more significant becomes the influence of such artifacts. Large peaks of the ECG signals were clearly observed during walking motion, but small peaks, such as P and T waves, were not distinguishable. During running motion, the ECG signal was totally distorted and indistinguishable when using commercial Ag/AgCl electrodes. However, as shown in Figure 9c, if P-electrodes are employed the ECG signal is very clear and stable for all three levels of body motion. We performed this experiment for three different subjects, but because similar results were measured from other subjects, only one result is depicted in Figure 9 (see Figures S1 and S2 in supplementary materials for the data from other subjects).

**Figure 10:**
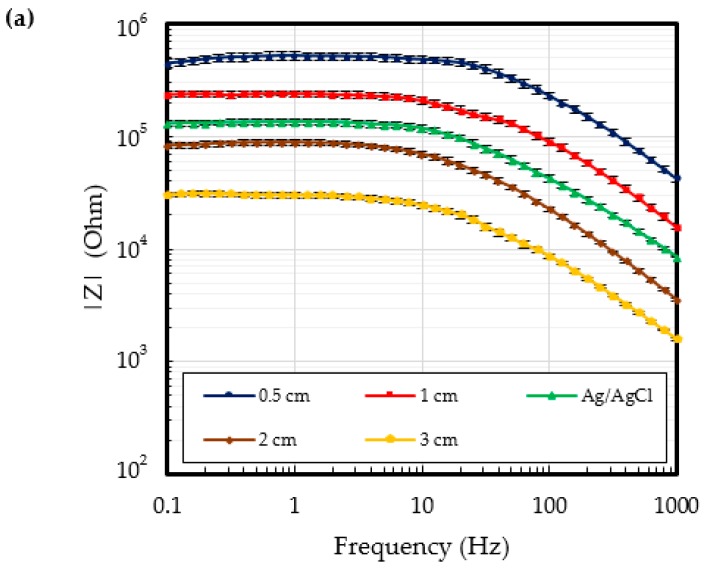

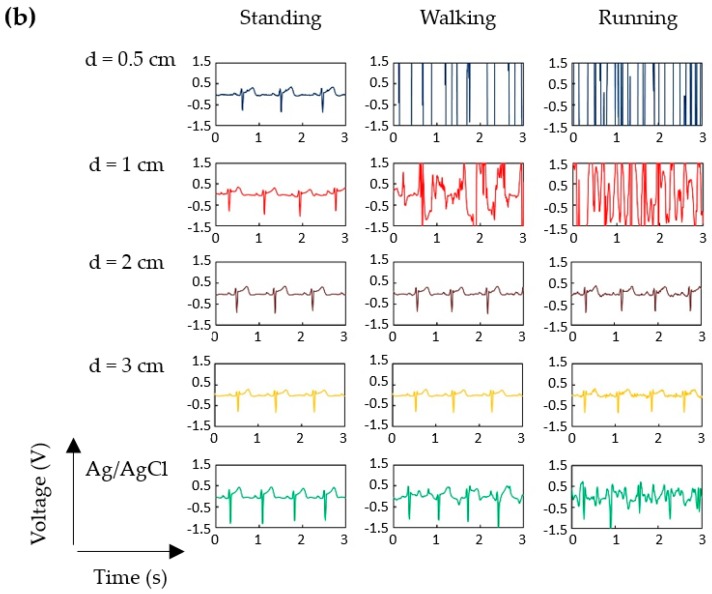
(**a**) Contact impedance and (**b**) ECG signals measured for P-electrodes of different diameters.

Figure 10 shows the contact impedances and the ECG signals measured for various dimensions of the P-electrodes applied to the skin. The diameter of silver fabric patch was of 10 mm for all experiments. As shown in Figure 10a, the lowest contact impedance was obtained when using the largest P-electrode. Noteworthy, if the diameter of P-electrode is larger than 20 mm, its contact impedance becomes lower than the commercial Ag/AgCl electrode. As shown in Figure 10b, if a P-electrode larger than 20 mm is employed, a stable ECG signal is obtained even when the subjects walk or run. Therefore, by increasing the diameter of P-electrodes, a high-quality ECG signal is obtained as a result of low contact impedance.

**Figure 11:**
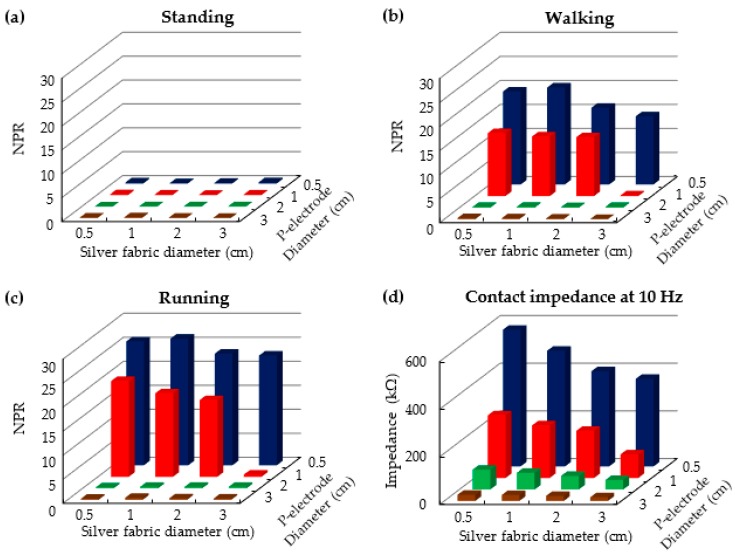
(**a**–**c**) Noise to P-wave ratio (NPR) in ECG signals obtained during three levels of motion for combinations of P-electrodes on the skin and silver fabric patches on the garment having different diameters; and (**d**) contact impedance measured at 10 Hz for the various combinations of silver fabric patches and P-electrodes.

Figure 11a–c depicts the noise to P-wave ratio (NPR) for different combinations of the dimensions of P-electrodes on the skin and silver fabric patches on the garment. The NPR was calculated by the magnitude of noise per magnitude of the P-wave. The P-wave is the smallest signal in an ECG waveform, and it is difficult to analyze the ECG signal if the noise is larger than the P-wave. In this study, the magnitude of the P-wave was defined as the average magnitude of P-waves obtained during the standing state. Noise is defined as the standard deviation of the fluctuating signal peaks between T- and P-wave peaks. In this experiment, we tested four different diameters (5, 10, 20, and 30 mm) for both the P-electrode and silver fabric patch. The NPR was very low when the subject was at rest, regardless of the electrode dimension. During both walking and running, the NPR was very small regardless of the dimension of silver fabric if the diameter of the P-electrode was larger than 20 mm. If the diameter of the P-electrode is 10 mm, the silver fabric patch needs to be larger than 30 mm in order to obtain a clear ECG signal. However, obtaining a good ECG signal becomes difficult if the diameter of the P-electrode is smaller than 5 mm. Figure 11d shows the contact impedance measured at 10 Hz for the various combinations of silver fabric patches and P-electrodes. The NPR is closely related to the contact impedance between the electrodes and the skin. In this experiment, a clear ECG signal was obtained without motion artifacts regardless of human motion when the contact impedance was lower than 99 kΩ. This experiment does not provide a generalized threshold impedance value to obtain a clear ECG signal because the threshold value was different among the subjects, skin conditions, and contact position on the skin, etc. in our experiment. However, it is clear that the motion artifact is closely related to the contact impedance of the electrodes on the skin, and can be minimized by adapting our proposed P-electrode.

**Figure 12:**
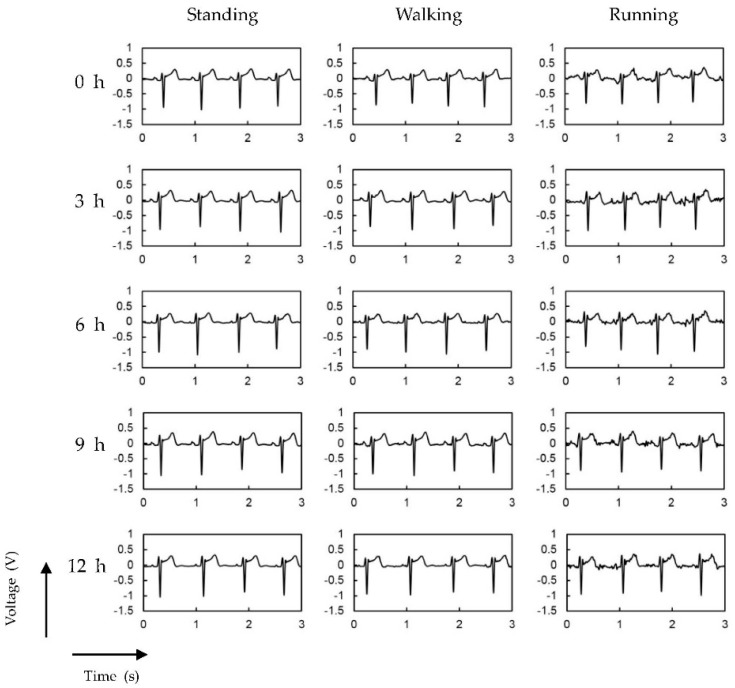
ECG signals measured every 3 h for 12 h after application of P-electrodes on skin.

We also tested the long-term stability up to 12 h of the proposed P-electrodes. Figure 12 shows the ECG signals measured every 3 h after the P-electrodes are placed on the skin. Additionally, the ECG signals obtained from commercial Ag/AgCl electrodes are shown in Figure S3. In the case of Ag/AgCl electrodes, the signal quality degrades, especially for walking and running cases, because of the drying of the electrolyte gel over time. When P-electrodes were used, stable ECG signals were measured for 12 h.

**Figure 13:**
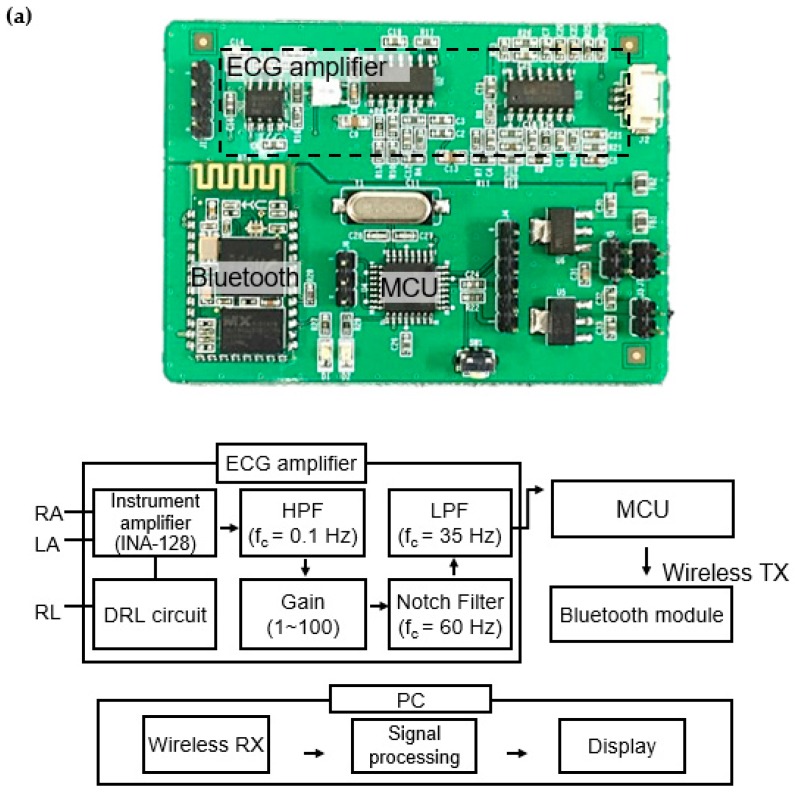

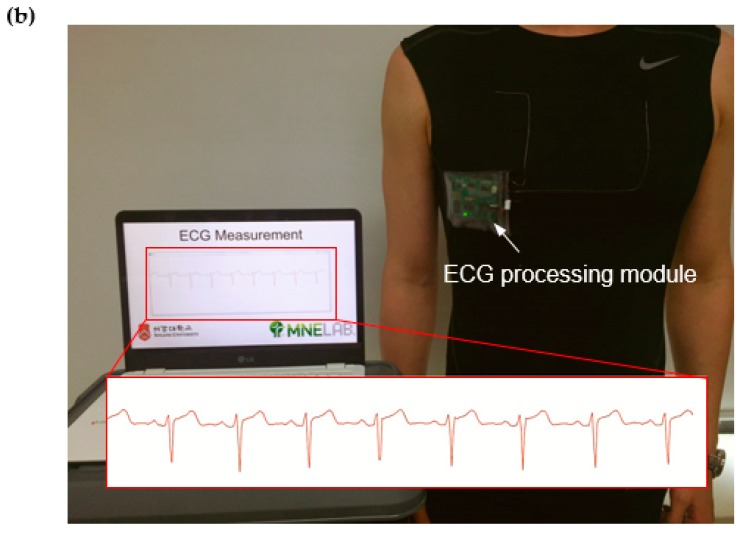
(**a**) ECG processing module and its functional block diagram; and (**b**) ECG measurement garment with the ECG processing module and the wirelessly-transmitted ECG signal. MCU: Microcontroller, RA: Right arm, LA: Left arm, RL: Right leg, DRL: Driven Right Leg, HPF: High pass filter, LPF: Low pass filter, TX: Transmitter, PC: Personal computer, RX: Receiver.

A wireless ECG processing module shown in Figure 13 was developed to measure ECG signals wirelessly by using the proposed ECG garment. The module consisted of an ECG amplifier, microprocessor, and Bluetooth module. Figure 13a shows the block diagram of the ECG processing module. The ECG signals are differentially amplified by an instrumentation amplifier (INA128, Texas Instruments, Dallas, TX, USA) and filtered by a high-pass filter (f_c_ = 0.1 Hz) to cancel the offset. The signals are then amplified and filtered again to reduce power line noise by means of a notch filter (f_c_ = 60 Hz) and a low-pass filter (f_c_ = 35 Hz). Finally, the signals are digitized by the microprocessor (ATmega328, power consumption: 5.1 mW at 3 V, Atmel, San Jose, CA, USA) and transmitted to a computer through a Bluetooth module (HC-06, power consumption: 26.4 mW at 3.3 V, Atomic Market, Waxahachie, TX, USA). The dimension of the module is 5 cm × 6.5 cm. The power is provided by a 7.4-V lithium-polymer battery. Figure 13b shows the ECG measurement garment and the clear ECG signals wirelessly transmitted from the subject’s body to the monitoring computer in real-time. No signal loss or degradation was found in the transmitted signals.

## 4. Conclusions

In this study, we propose and demonstrate a new ECG measurement garment that employs flexible patch electrodes made of conductive carbon paste. The P-electrodes adhere to the skin to reduce the motion artifacts. The proposed P-electrodes are easy to fabricate and remove after use. The experiments confirmed that the measured contact impedance using P-electrode was lower than that of commercial Ag/AgCl electrodes because the P-electrode achieves conformal contact with the skin. P-electrodes provide stable and excellent ECG signals even when the subject is walking or running. In this experiment, the P-electrode shows a stable ECG signal up to 12 h after application to the skin. Further experiment and study are required for stability and reliability. In addition, skin safety is an important issue, because the P-electrode is directly applied to human skin. In this experiment, no severe skin problems were monitored, except for slight reddening and irritation. However, further long-term clinical studies are required before practical usage of the proposed device is advised. In this study, P-electrodes were used to measure ECG signals, but we expect they can also be used for monitoring other bio-signals, such as EEG and EMG.

## Supplementary Materials

The following are available online at www.mdpi.com/2073-4360/9/9/439/s1, Figure S1: Measured ECG signal at three different levels of motion (standing, walking, and running) for a second subject by using (a) ECG measurement garment without P-electrodes, (b) Ag/AgCl electrodes, and (c) ECG measurement garment with P-electrodes; Figure S2: Measured ECG signal at three different levels of motion (standing, walking, and running) for a third subject by using (a) ECG measurement garment without P-electrodes, (b) Ag/AgCl electrodes, and (c) ECG measurement garment with P-electrodes; Figure S3: ECG signals measured every 3 h for 12 h after application of commercial Ag/AgCl electrodes on skin.
