# Supplementary material for: ECG Monitoring Garment Using Conductive Carbon Paste for Reduced Motion Artifacts
Source: Polymers (Basel). 2017 Sep 11;9(9):439. doi: 10.3390/polym9090439 (PMC6418715; doi:10.3390/polym9090439)
Supplement: Supplementary file 1 [file polymers-09-00439-s001.pdf]

polymers

MDPI

Supplementary Materials: ECG Monitoring Garment Using Conductive Carbon Paste for Reduced Motion Artifacts

Jin-Woo Lee and Kwang-Seok Yun

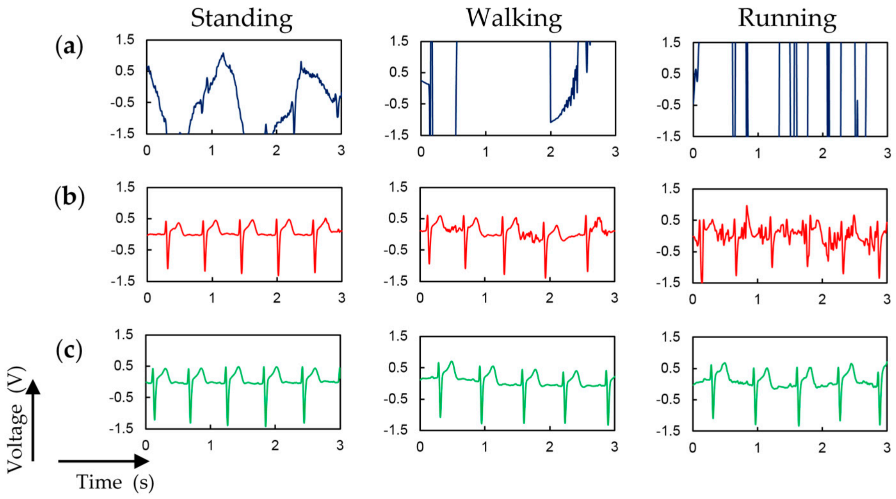

Figure S1. Measured ECG signal at three different levels of motion (standing, walking, and running) for second subject by using (a) ECG measurement garment without P-electrodes, (b) Ag/AgCl electrodes, and (c) ECG measurement garment with P-electrodes.

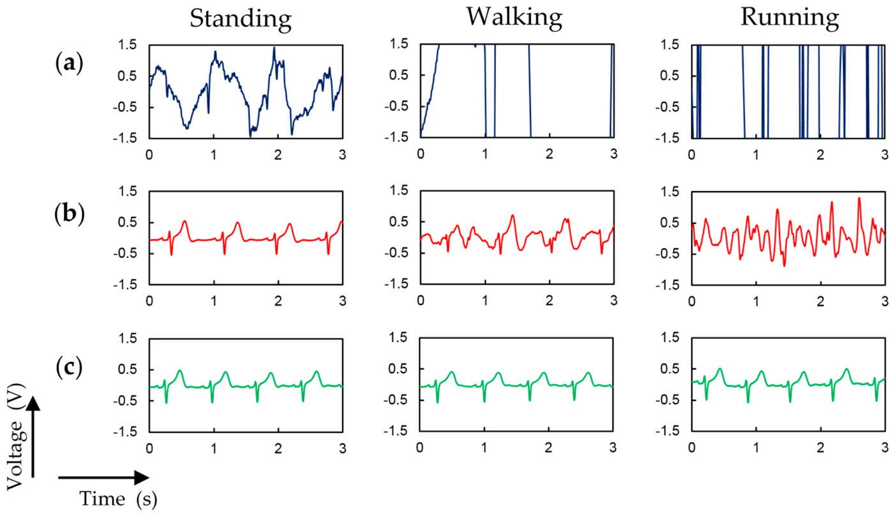

Figure S2. Measured ECG signal at three different levels of motion (standing, walking, and running) for third subject by using (a) ECG measurement garment without P-electrodes, (b) Ag/AgCl electrodes, and (c) ECG measurement garment with P-electrodes.

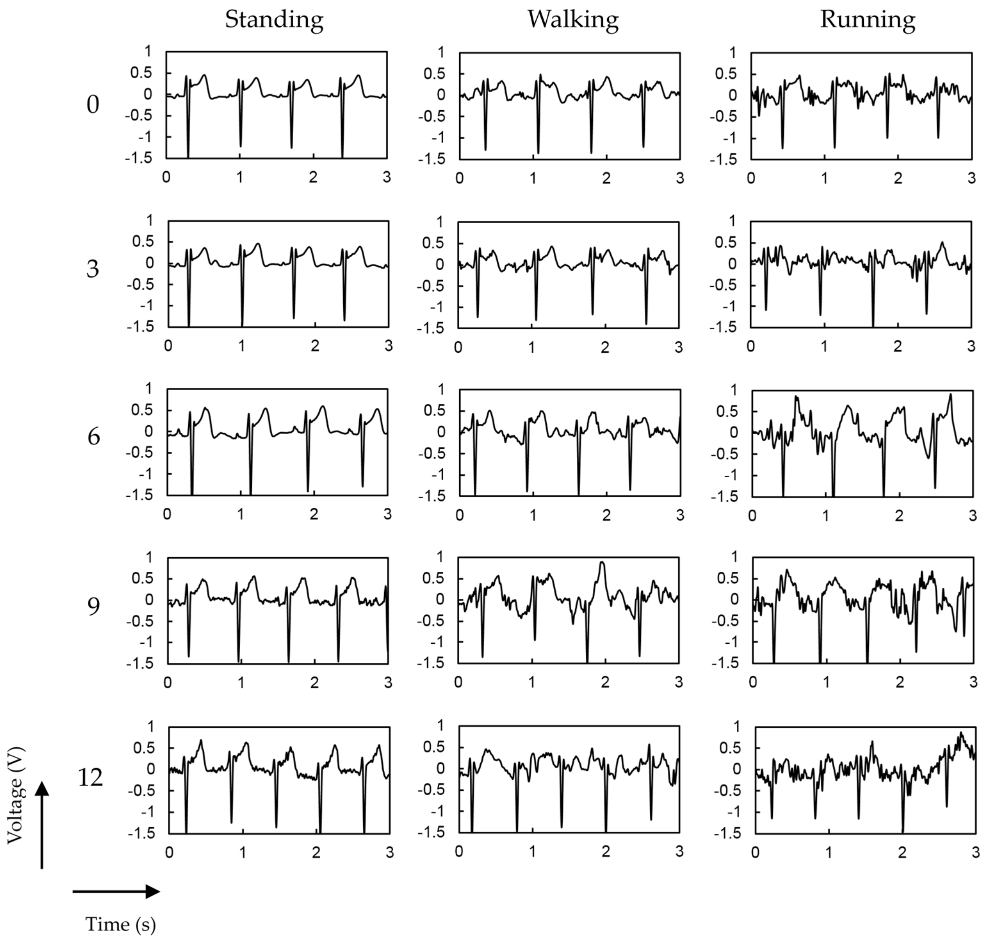

Figure S3. ECG signals measured every 3 h for 12 h after application of commercial Ag/AgCl electrodes on skin.
